# Symptoms of anxiety, depression and post-traumatic stress in pairs of patients and their family members during and following ICU stay: who suffers most?

**DOI:** 10.1186/cc13215

**Published:** 2014-03-17

**Authors:** R Fumis, O Ranzani, P Martins, G Schettino

**Affiliations:** 1 Hospital Sírio Libabês, Sao Paulo, Brazil; 2 Hospital das Clínicas, Universidade de Sao Paulo, Brazil

## Introduction

To compare the incidence of anxiety, depression and post-traumatic stress symptoms in pairs of patients and respective family members during the ICU stay and at 30 and 90 days post ICU discharge. According to the literature, both patients and family members suffer from psychological distress during and following an ICU stay [1]. Although these issues have been discussed, to date few studies have addressed the pairs at three times.

## Methods

A prospective study conducted in a 22-bed adult general ICU including patients staying >48 hours. The Hospital Anxiety and Depression Scale (HADS) was completed by pairs (patients/respective family members). They were interviewed by telephone at 30 and 90 days after ICU discharge with the Impact of Event Scale (IES) and the HADS. We separated them into three different groups (patients - group A, family member of patient who survived - group B and family member of patient who deceased - group C).

## Results

A total of 184 pairs were interviewed at the ICU. Mean patient age was 59.33 ± 15.5 years. The admission SAPS III was 47.6 ± 15.7 points. Median ICU lOs was 4 days (2 to 47). Family's age was 51.79 ± 13.47 and 64% were spouses. Patients had symptoms of anxiety (26.1%), depression (12%) or both (8.7%) during the ICU stay. Regarding family members, the incidence of anxiety, depression or both during the ICU was 33.2%, 9.8% and 8.2% respectively. Symptoms of PTSD within patients were found in 6.9% at 30 days and disappeared at 90 days. Differently, these symptoms in family members were found in 6.2% at 30 days and 9.0% at 90 days. There was a positive correlation between HADS at ICU and IES at 30 days for family members (*r *= 0.527, *P *< 0.001). The HADS score over time between patients, family members of patients who deceased and who survived were different (*P *= 0.002). In *post-hoc *analysis, group A presented less symptoms than group C at 30 days (*P *= 0.001) and less than groups B and C at 90 days (*P *< 0.001 for both). At 90 days, group B showed less symptoms than group C (*P *= 0.039).

## Conclusion

Family members of ICU patients suffer more than the patients principally when their loved one died. Patients' symptoms of anxiety, depression and PTSD decrease with time but in family members these symptoms continue along the 90 days.

## References

[B1] Myhren H Patients' memory and psychological distress after ICU stay compared with expectations of the relatives Intensive Care Med 2009 35 2078 2086 10.1007/s00134-009-1614-1 19756511

